# Effect of dietary supplementation with *Brevibacillus laterosporus* on broiler growth performance, meat quality and gut microbiome

**DOI:** 10.3389/fmicb.2025.1608076

**Published:** 2025-06-18

**Authors:** Haixia Han, Li Fu, Jie Wang, Yan Sun, Dingguo Cao, Qiuxia Lei, Yan Zhou, Fuwei Li, Wei Liu, Dapeng Li, Dan Hao, Jie Liu

**Affiliations:** ^1^Poultry Research Institute, Shandong Academy of Agricultural Sciences, Ji’nan, China; ^2^Main Animal Breeding Key Laboratory of Shandong Province, Ji’nan, China; ^3^Poultry Breeding Engineering Technology Center of Shandong Province, Ji’nan, China; ^4^JiNan Key Laboratory of Poultry Germplasm Resources Innovation and Healthy Breeding, Ji’nan, China

**Keywords:** *B. laterosporus*, broilers, growth performance, meat quality, antioxidant capacity, gut microbiota

## Abstract

This study aimed to investigate the effects of *Brevibacillus laterosporus* (*B. laterosporus*) supplementation on growth performance, carcass traits, antioxidant capacity, and cecal microbiota in broilers. A total of 320 one-day-old YS909 male broilers were randomly assigned to four dietary groups: control (CON), low-dose (LBL, 100 mg/kg), medium-dose (MBL, 300 mg/kg), and high-dose (HBL, 500 mg/kg) *B. laterosporus* supplementation (*n* = 8 replicates/group, 10 chicks/replicate). Growth performance, carcass traits, antioxidant capacity, and cecal microbiota/metabolites (MBL vs. CON) were analyzed. Dietary supplementation with *B. laterosporus* significantly decreased the feed intake / weight gain (F/G) in LBL (22–42 d and 1–42 d), MBL (22–42 d) and HBL (22–42 d) groups. Both MBL and HBL groups showed higher semi-eviscerated percentages than the control. The MBL group had a significantly increased eviscerated percentage. The LBL group had a significantly increased breast muscle percentage. Specifically, the HBL group exhibited a notable increase in muscle C18:3n3 content, and a significant decrease in muscle C18:1n9t and C20:3n3 content. The LBL group saw significant reductions in the proportion of C18:1n9t and C20:1. Additionally, the MBL group experienced significant decreases in the proportions of C18:3n3, C20:3n3 and C20:4n6. Dietary supplementation with *B. laterosporus* significantly enhanced the oxidative stress resistance of serum by decreasing malondialdehyde (MDA) levels and increasing glutathione peroxidase (GSH-PX) and total antioxidant capacity (T-AOC). 16S ribosomal DNA and metabolome sequencing of cecum contents was conducted for the MBL and CON groups. This analysis demonstrated significant increases in α-diversity indices in the MBL group. There was an increased relative abundance of *Firmicutes* and a decreased relative abundance of *Bacteroidetes* and *Proteobacteria* in the MBL group. In addition, the shifts of the cecal microbial community lead to the alteration of metabolites of the cecum including amino acid and lipid. In conclusion, dietary supplementation with medium-dose *B. laterosporus* enhanced broiler carcass traits and antioxidant status by modulating cecal microbiota and metabolites, demonstrating its potential as an effective feed additive.

## 1 Introduction

Intensive poultry production plays a crucial role in sustaining the nutritional needs of an expanding global population. However, intensive poultry production has led to frequent stress and disease outbreaks in the animals. Hence, the global use of antibiotic growth promoters has been prevalent for preventing infectious diseases in poultry. However, the chronic overuse or incorrect application of these antibiotics has led to the occurrence of antibiotic residues in poultry products, resistance to antibiotics among bacteria, and a disruption in the balance of microflora (Muhammad et al., 2020; Ma et al., 2021; Tian et al., 2021). This issue has caused numerous countries to implement bans on the use of antibiotics. Consequently, exploring alternative solutions to antibiotics has turned into a primary area of research within the poultry industry. Probiotics have demonstrated significant potential in poultry production, with documented benefits including improved microflora balance, enhanced intestinal structure, strengthened immunity, and optimized growth performance (Halder et al., 2024). Research has shown that *Lactobacillus acidophilus* supplementation in broilers significantly improves feed efficiency (*p* < 0.05) while reducing abdominal fat deposition, with a notable trend toward enhanced humoral immunity (IgG, *p* = 0.093) (Liu et al., 2025). Similarly, *Bacillus subtilis* has been shown to effectively modulate the gut microbiota in laying hens, producing significant anti-inflammatory effects, strengthening duodenal barrier integrity, and improving systemic antioxidant capacity (Zou et al., 2022). For broilers facing mycotoxin challenges, *Bacillus licheniformis* supplementation has proven particularly effective in counteracting both mycotoxin-induced enterotoxicity and necrotic enteritis pathology (Jamil et al., 2025). Furthermore, *Lactobacillus plantarum* supplementation has demonstrated dual benefits, significantly improving growth performance while simultaneously elevating immune organ indices and serum IgA and IgG levels (*P* < 0.05) (Wang et al., 2023).

*Brevibacillus laterosporus* is a Gram-positive bacterium that produces spores in nature. It is found in soil, fresh water, sea water, insect bodies, and plant surfaces. *B. laterosporus*, which produces a variety of metabolites, including antimicrobial peptides and enzymes, exhibits a wide range of antimicrobial activity against bacteria, fungi, and protozoa (Liu Y. et al., 2024). It is widely used as a biological control agent for plant pathogens. Emerging evidence highlights the multifaceted benefits of *B. laterosporus* in poultry production. *B. laterosporus* exhibits potent antimicrobial activity against key pathogens, including *Escherichia coli K88* and *Salmonella typhimurium ATCC 14028* (Che et al., 2016; Purba et al., 2022), while simultaneously enhancing growth performance through improved feed conversion efficiency and body weight gain (Wolfenden et al., 2011). Dietary supplementation with *B. laterosporus strain S62-9* significantly enhanced broiler performance, demonstrating a 7.2% increase in body weight and a 5.19% reduction in feed conversion ratio, while concurrently improving immune parameters (Zhi et al., 2024). Additionally, supplementation with *B. laterosporus S62-9* positively influenced meat quality, as evidenced by elevated pH, improved brightness, and increased tenderness (Liu et al., 2023). These collective findings position *B. laterosporus* as a promising multifunctional feed additive for modern broiler production systems, offering both antimicrobial protection and performance-enhancing properties.

This study investigated the effects of varying doses of *B. laterosporus* on production performance, carcass trait, meat quality, and antioxidant capacity in YS909 broilers. Furthermore, we employed an integrated approach combining metabolomics with 16S rDNA sequencing to characterize its effects on cecal microbiota composition and metabolic profiles. The results offer evidence-based recommendations for optimizing *B. laterosporus* application in broiler production systems.

## 2 Materials and methods

### 2.1 Microbial agent preparation

*B. laterosporus* was inoculated from an agar slant culture stored at 4°C into nutrient broth medium for activation (12 h at 36°C, 200 rpm). Afterward, the strain was fermented in a high-yield optimized medium for 48 h (36°C, 200 rpm) and subsequently spray-dried into a powdered microbial agent containing *B. laterosporus* at a concentration of 5 × 10^10^ CFU/g.

### 2.2 Experimental design and animal management

This research involved 320 male YS909 broilers, each aged 1 day, with closely matched starting weights. These chicks were randomly assigned into four primary groups, with each group comprising eight smaller units, each unit housing 10 chicks. Throughout the duration of this study, the chicks were subjected to an unvarying lighting schedule. They had continuous access to both mash feed and fresh water. The nutritional details of their diet are presented in Supplementary Table S1. The groups were: (1) a basal diet, control group (CON); (2) a basal diet + 100 mg/kg of *B. laterosporus* (>5 × 10^10^CFU/g), a low-dose of *B. laterosporus* group (LBL); (3) a basal diet + 300 mg/kg of *B. laterosporus*, a medium-dose of *B. laterosporus* group (MBL); and (4) a basal diet + 500 mg /kg of *B. laterosporus*, a high-dose of *B. laterosporus* group (HBL). The experiment lasted 42 d with two feeding periods. The first period was from day 1 to 21, and the second period was from day 22 to 42.

### 2.3 Growth performance

We recorded the weight of the broilers per cage and monitored their feed intake on the first, 21st and 42nd days. This data was used to determine the feed intake/weight gain (F/G), along with the average daily feed intake (ADFI) and average daily gain (ADG).

### 2.4 Sample collection and index determination

On the 42nd days, we selected eight birds from each group, each bird representing a replicate, ensuring their weights were closely matched. We then collected blood samples from the wing vein of these birds, which were subsequently centrifuged at 4,000 rpm for 15 min. After centrifugation, we separated the serum and preserved it at a temperature of –80°C for later analysis of antioxidant. Post blood collection, the birds were humanely euthanized through electrical stunning and exsanguination. In accordance with NY/T 823-2,004 standards, dissections were performed on each carcass to evaluate a range of metrics, including dressing, semi-eviscerated, and eviscerated percentages, in addition to the ratios of breast muscle to abdominal fat. Calculation of these metrics utilized specific formulas: The dressing percentage was derived by the formula (carcass weight/total body weight) × 100. Similarly, semi-eviscerated and eviscerated percentages were calculated as (weight of semi-eviscerated or eviscerated carcass/body weight) × 100. The proportion of breast muscle was ascertained by (breast muscle weight/eviscerated weight) × 100, and the abdominal fat percentage was determined by [abdominal fat weight/(eviscerated weight + abdominal fat weight)] × 100. For further investigation, liver and breast muscle samples were collected. Additionally, cecal chyme from the CON and MBL groups was promptly frozen in liquid nitrogen and stored at –80°C for subsequent 16S rDNA and metabolome analysis.

### 2.5 Antioxidant indices analysis

The muscle or liver were prepared by immersing it in a 1:9 w/v cold saline solution (1 g of tissue per 9 mL of PBS), followed by homogenization in ice and centrifugation at 12,000 rpm for 15 min. The supernatant obtained was then used for determining the antioxidant capacity. The serum, muscle and liver antioxidant indices were analyzed using an ELISA kit (Bioswamp, Wuhan, China)(The detection sensitivities are as follows: SOD, 0.1 pg/mL; GSH-PX, 0.1 ng/mL; TAOC, 0.01 U/mL; CAT, 0.1 pg/mL; MDA, 0.01 mmol/L).

### 2.6 Meat quality and fatty acid composition analysis

The evaluation of meat quality involved analyzing the pH levels, color metrics, and shear force of the breast muscle. A portable pH meter (Testo 205, Testo AG, Germany) facilitated the pH measurement in the breast muscle, taken 45 min after the animals demise. Color parameters—lightness (L*), redness (a*), and yellowness (b*)—were ascertained with a Chroma Meter (NR10QC, 3nh, China), adhering to the instructions provided by its manufacturer. For thermal processing, a 20-gram section of the muscle was weighed and submerged in a water bath until it reached a core temperature of 75°C. Subsequently, this portion was sectioned into strips measuring 2 cm in length, 1 cm in width, and 1 cm in height, oriented parallel to the muscle fibers. A digital texture tenderness analyzer (C-LM3B, TenovoFood, China) was employed to assess the shear force. The composition of muscle fatty acids was assessed using the following procedure: 80 mg of freeze-dried powder samples were precisely measured and placed into a hydrolysis tube. To the sample, 200 μl of CH3(CH2)15COOH-C6H14 (5 mg/mL) was added, followed by the addition of 4 ml of acetyl chloride solution. The mixture was then subjected to nitrogen protection and agitation. Subsequently, it was agitated every 20 min while being heated in an 80°C water bath for 2 h. Upon cooling to ambient temperature, 4 ml of n-hexane was added for further agitation, followed by the addition of 8 ml of 6% K2CO_3_ for 5 min of agitation. The organic phase was then transferred to a 15 ml centrifuge tube and centrifuged at 4,000 rpm for 10 min. The supernatant organic layer was filtered through a 0.22 μm organic filter membrane and analyzed by gas chromatography (TRACE 1,310, Thermo Fisher, Germany), in accordance with the manufacturer’s guidelines.

### 2. 7 Microbial composition analysis

#### 2.7.1 DNA extraction

Using the CTAB method (Andreou, 2013), we extracted DNA from various samples. This reagent, known for its efficacy in isolating DNA even from minimal sample quantities, has proven successful in preparing bacterial DNA. We employed nuclear-free water as a control blank. Subsequently, the extracted total DNA was eluted using 50 μL of elution buffer and then preserved at –80°C. This step was carried out before proceeding to PCR amplification, which was conducted by LC-Bio Technology Co., Ltd. (Hangzhou, China).

#### 2.7.2 PCR amplification and 16S rDNA sequence

We determined the diversity of the microflora by sequencing the V3-V4 region of the 16S rDNA gene, amplified previously. The primers used were: forward primer of the 16S amplicon PCR, 5′-CCTACGGGNGGCWGCAG-3′, and its reverse counterpart, 5′-GACTACHVGGGTATCTAATCC-3′ (Logue et al., 2016). The PCR amplification utilized a reaction mix of 25-μL, comprising 25 ng of template DNA, 12.5 μL of PCR premix, 2.5 μL of each primer, and PCR-grade water. Our protocol for amplifying prokaryotic 16S fragments included a starting denaturation at 98°C for 30 s, followed by 32 cycles of denaturation at 98°C for 10 s, annealing at 54°C for 30 s, and extension at 72°C for 45 s. This process concluded with a final extension at 72°C for 10 min. We conducted 2% agarose gel electrophoresis to confirm the PCR products. To avoid false-positive PCR outcomes, ultrapure water served as the negative control during DNA extraction. Post-PCR, we purified the products using AMPure XT beads (Beckman Coulter Genomics, Danvers, MA, United States) and quantified them with a Qubit (Invitrogen, United States). For sequencing, we pooled the amplicons and evaluated the library’s size and quantity using an Agilent 2100 Bioanalyzer (Agilent, United States) and a Library Quantification Kit for Illumina (Kapa Biosciences, Woburn, MA, United States). The NovaSeq PE250 platform was employed for sequencing.

#### 2.7.3 Data analysis

On the Illumina NovaSeq platform, as per the LC-Bio protocol, we executed the sequencing of the samples. This involved allocating unique barcodes to pair-end reads for each sample, and subsequently, these barcodes and primer sequences were removed. The merging of these pair-end reads was performed using FLASH (Magoč and Salzberg, 2011). For assessing the quality of the initial reads, we employed fqtrim (v0.94) with specific filtering criteria to produce high-quality clean tags (Edwards, 2011). Chimeric sequences were removed using Vsearch software (v2.3.4) (Rognes et al., 2016). Using DADA2, we achieved the dereplication process, resulting in both a feature sequence and a feature table (Callahan et al., 2016). Random sequencing normalization was the method used for normalizing alpha diversity. We adjusted the feature abundance based on each sample’s relative abundance, following the SILVA (release 138) classifier guidelines (Quast et al., 2013). Our alpha diversity analysis, which assessed species diversity complexity, employed four indices: Chao1, observed species, Shannon, and Simpson, using QIIME2 for calculation (Bolyen et al., 2019). We aligned the sequences via Blast and used the SILVA database for annotating the feature sequences. The abundance analysis of each species in every sample was based on the ASV abundance table, with a set confidence threshold for annotations at 0.7. To identify biological differences between groups, LEfSe was employed. Furthermore, we used the Mann Whitney U test for analyzing differences between the MBL and CON groups based on species abundance statistics.

### 2.8 Metabolomics sequencing

#### 2.8.1 Sample treatment

A 100 mg sample was extracted with 1 mL of precooled 50% methanol, vortexed for 1 min, and incubated at room temperature for 10 min. The extract was then stored at –20°C overnight. Following centrifugation at 4,000 × g for 20 min, the supernatant was transferred to a new 96-well plate.

#### 2.8.2 Metabolite analysis by LC–MS/MS

All samples were analyzed using the LC-MS system according to the manufacturer’s instructions. Chromatographic separations were performed using the UltiMate 3000 UPLC system (Thermo Fisher Scientific, Bremen, Germany). Reverse-phase separation was carried out on an ACQUITY UPLC T3 column (100 mm × 2.1 mm, 1.8 μm; Waters, Milford, United States). Metabolites eluted from the column were analyzed using a high-resolution tandem mass spectrometer, the TripleTOF 6600 (SCIEX, Framingham, MA, United States). To ensure the stability of the LC-MS system throughout the analysis, a quality control sample (a pooled mixture of all samples) was injected after every 8 experimental samples.

#### 2.8.3 Data analysis

Mass spectrometry data were pre-processed using XCMS software, including peak detection, peak grouping, retention time correction, secondary peak grouping, and annotation of isotopes and adducts (Smith et al., 2006). Metabolites were annotated by matching the exact molecular mass (m/z) of the samples with entries in the KEGG and HMDB databases. Metabolites with a mass difference of less than 10 ppm between the observed and database values were annotated, and their molecular formulas were further confirmed using isotope distribution patterns.

Statistical analysis was performed using R software (version 4.0). Raw intensity values were normalized using median normalization, and significantly differentially expressed metabolites were identified using the R package metaX (Wen et al., 2017). Partial least squares discriminant analysis (PLS-DA) was conducted using the R package ropls, and variable importance in projection (VIP) scores were calculated for each metabolite. Correlation analysis was performed using Pearson’s correlation coefficient via the R cor function. Metabolites were considered significantly different if they simultaneously met the following criteria: (1) P-value < 0.05 from a *t*-test, (2) fold change > 1.2, and (3) VIP score > 1 from PLS-DA analysis. Pathway enrichment analysis of KEGG pathways was performed using a hypergeometric test, with pathways showing a *P* < 0.05 considered significantly enriched.

### 2.9 Statistical analysis

The analysis of growth performance, antioxidant capacity, fatty acids content and meat quality statistics was conducted utilizing SPSS 22.0, specifically designed for Windows (SPSS Inc., Chicago, IL). We employed a one-way ANOVA and subsequently used the LSD multiple comparison test to assess the variations between the different groups.

In ANOVA, each subject’s score is based on the following equation:


$$y_{i\,j}=u+\tau_{j}+e_{i\,j}$$

where y_*ij*_ represents the score of the i^th^ participant in the j^th^ group, μ the grand population mean, τ_*j*_ the treatment applied to the j^th^ group, and e_*ij*_ the error associated with the i^th^ participant in the j^th^ group.

## 3 Results

### 3.1 Growth performance and carcass traits

Table 1 shows growth performance. During the first period (up to 21 d of age), the CON group had lower F/G than the HBL group (*P* < 0.01). During the second period (22–42 d of age), the LBL, MBL and HBL groups had lower F/G than the CON group (*P* < 0.05), and the LBL and HBL groups had lower ADFI than the CON group (*P* < 0.05). Over the whole experimental period, the LBL group had lower F/G (*P* < 0.05) than the CON group, while the BW42, BW21, ADG were not significantly changed by supplementation with *B. laterosporus*.

**TABLE 1:** Effect of *B. laterosporus* supplementation on growth performance of broilers.

Item	Group				SEM	*P*-value
	CON	LBL	MBL	HBL		
BW1	44.16^b^	44.82^ab^	44.76 ^ab^	45.57^a^	0.43	0.20
BW21	438.11	424.20	435.60	422.55	11.50	0.37
BW42	1279.34	1249.94	1323.75	1242.90	15.60	0.25
ADFI1-21	27.44	27.35	28.80	29.04	1.21	0.37
ADG1-21	18.76	18.06	18.61	17.95	0.56	0.40
F/G1-21	1.46^Bb^	1.51^ABb^	1.55^ABab^	1.62^Aa^	0.05	0.02
ADFI22-42	78.84^a^	71.23^b^	78.10^a^	70.84^b^	2.97	0.01
ADG22-42	40.06	39.32	42.29	39.06	1.77	0.27
F/G22-42	1.97^a^	1.82^b^	1.85^b^	1.82^b^	0.06	0.05
ADFI1-42	53.14^ab^	49.29^b^	53.45^a^	49.94 ^ab^	1.92	0.08
ADG1-42	29.41	28.69	30.45	28.51	1.03	0.25
F/G1-42	1.81^a^	1.72^b^	1.76 ^ab^	1.75 ^ab^	0.04	0.16

ADFI, average daily feed intake; ADG, average daily weight gain; BW, body weight; F/G, feed intake / weight gain; CON, control group; HBL, 500 mg/kg group; LBL, 100 mg/kg group; MBL, 300 mg/kg group. ^A,B^ Means of a row with no common superscript are significantly different (*P* < 0.01). ^a,b^ Means of a row with no common superscript are significantly different (*P* < 0.05).

Table 2 demonstrates the effects of *B. laterosporus* dietary supplementation on broiler chicken carcass traits. Relative to the control group, an elevation in the semi-eviscerated percentage was observed in both MBL and HBL groups (*P* < 0.05). In addition, eviscerated percentage was increased in MBL group compared to the CON group (*P* < 0.01). Breast muscle percentage was increased in LBL group compared to the CON group (*P* < 0.05). Nonetheless, there were no significant differences in dressing percentage or abdominal fat percentage among the various treatment groups.

**TABLE 2:** Effects of *B. laterosporus* supplementation on carcass traits of broilers.

Item	Group				SEM	*P*-value
	CON	LBL	MBL	HBL		
Dressing percentage (%)	90.18	90.07	90.22	90.31	0.14	0.94
Semi-eviscerated percentage (%)	81.34^b^	81.24^b^	83.32^a^	83.23^a^	0.35	0.03
Eviscerated percentage (%)	66.55^BC^	65.28^C^	69.26^A^	68.05^AB^	0.42	0.01
Breast muscle (%)	19.18^ABb^	20.53^Aa^	19.47^ABb^	18.73^Bb^	0.21	0.01
Abdominal fat (%)	2.15	3.07	2.66	2.43	0.18	0.32

^A−C^Means of a row with no common superscript are significantly different (*P* < 0.01). ^a,b^Means of a row with no common superscript are significantly different (*P* < 0.05).

### 3.2 Meat quality assessment

Table 3 illustrates the effect of *B. laterosporus* supplementation on broiler chicken meat quality. Supplementation did not significantly impact shear force, pH, or color indicators, including Lightness (L*), Redness (a*), and Yellowness (b*).

**TABLE 3:** Effects of *B. laterosporus* supplementation on meat quality of broilers.

Item	Group				SEM	*P*-value
	CON	LBL	MBL	HBL		
Shear force (Kg)	2.60	2.64	2.62	2.89	0.13	0.86
45 min PH	5.78	5.70	5.70	5.72	0.25	0.60
L*	46.15	45.87	44.33	44.13	0.46	0.29
a*	2.71	1.72	3.11	1.94	0.28	0.26
b*	11.05	11.72	10.91	10.90	0.48	0.93

L*, lightness; a*, redness; b*, yellowness.

### 3.3 Fatty acid composition analysis

Table 4 presents the fatty acid composition in the breast muscle, revealing 22 fatty acids, consisting of 9 saturated fatty acids (SFA) and 13 unsaturated fatty acids (UFA). The most prominent fatty acids were palmitic acid (C16:0), stearic acid (C18:0), oleic acid (C18:1n9c), and linoleic acid (C18:2n6c). *B. laterosporus* supplementation had no significant effect on SFA levels in the breast muscle. In contrast, the unsaturated fatty acid, α-linolenic acid (C18:3n3), displayed a significant increase in the HBL group (*P* < 0.01), whereas elaidic acid (C18:1n9t) levels significantly decreased in both LBL (*P* < 0.05) and HBL groups (*P* < 0.01). Additionally, the proportions of eicosenoic acid (C20:1) significantly decreased in LBL group (*P* < 0.05), while the proportions of eicosadienoic acid (C20:3n3) (*P* < 0.01) and arachidonic acid (C20:4n6) (*P* < 0.05) significantly decreased in the MBL group, and the proportions of C20:3n3 also decreased in the HBL group (*P* < 0.01).

**TABLE 4:** Effects of *B. laterosporus* supplementation on fatty acids content in breast muscle of broilers (%).

Iterm	Group				SEM	*P*-value
	CON	LBL	MBL	HBL		
**Saturated fatty acid (SFA)**						
C13:0	1.09	1.23	1.19	1.04	0.03	0.14
C14:0	0.33	0.33	0.33	0.34	0.01	0.88
C15:0	0.09	0.08	0.09	0.09	0.00	0.91
C16:0	23.26	22.67	23.19	23.31	0.17	0.53
C17:0	0.28	0.29	0.24	0.28	0.01	0.63
C18:0	13.10	12.39	12.11	12.38	0.20	0.36
C20:0	0.11	0.11	0.12	0.11	0.01	0.96
C22:0	1.11	1.01	1.10	1.08	0.03	0.55
C23:0	0.56	0.56	0.54	0.62	0.02	0.44
**Unsaturated fatty acids (UFA)**						
C16:1	1.19	1.14	1.21	1.22	0.09	0.99
C18:1n9t	0.10^a^	0.07^b^	0.08^ab^	0.07^b^	0.00	0.07
C18:1n9c	18.38	18.70	18.40	18.79	0.37	0.98
C18:2n6c	25.89	26.53	26.69	26.22	0.38	0.90
C18:3n3	0.16^Bb^	0.15^Bbc^	0.12^Bc^	0.24^Aa^	0.01	0.00
C18:3n6	1.08	1.19	1.22	1.25	0.04	0.54
C20:1	0.18^ABa^	0.16^Bb^	0.19^Aa^	0.18^ABa^	0.00	0.03
C20:2	0.80	0.73	0.82	0.76	0.02	0.54
C20:3n3	0.22^A^	0.19^A^	0.09^B^	0.06^B^	0.01	0.00
C20:4n6	0.16^ABa^	0.15^ABab^	0.11^Bb^	0.17^Aa^	0.01	0.05
C20:5n3	1.00	1.00	0.99	0.96	0.03	0.96
C22:1n9	7.12	7.29	6.98	6.83	0.18	0.85
C22:6n3	3.80	4.04	4.20	4.02	0.10	0.54
SFA	39.92	38.66	38.91	39.24	0.28	0.43
MUFA	26.97	27.36	26.86	27.08	0.34	0.97
PUFA	33.11	33.98	34.24	33.68	0.38	0.77
UFA	60.08	61.34	61.09	60.76	0.28	0.43
P/S	0.83	0.88	0.88	0.86	0.01	0.57
n-3	5.17	5.39	5.40	5.28	0.11	0.89
n-6	27.93	28.59	28.84	28.40	0.41	0.90
n6/n3	5.49	5.36	5.55	5.42	0.18	0.99

C13:0, Tridecanoic acid; C14:0, Myristic acid; C15:0, Pentade-canoic acid; C16:0, Palmitic acid; C17:0, Heptadecanoic acid; C18:0, Stearic acid; C20:0, Arachidic acid; C22:0, Docosanoic acid; C23:0, Tricosanic acid; C16:1, Palmitoleic acid; C18:1n9t, Elaidic acid; C18:1n9c, Oleic acid; C18:2n6c, Linoleic acid; C18:3n3, α-Linolenic acid; C18:3n6, γ-Linolenic acid; C20:1 Eicosenoic acid; C20:2, Eicosadienoic acid; C20:3n3, Cis-11,14,17-Eicosatrienoate; C20:4n6, Arachidonic acid; C20:5N3, Timnodonic acid; C22:1n9, Erucic acid; C22:6n3, Docosahexaenoic acid; MUFA, monounsaturated fatty acid; PUFA, polyunsaturated fatty acid; SFA, Saturated fatty acid; P/S, PUFA/ SFA. ^A,B^Means of a row with no common superscript are significantly different (*P* < 0.01). ^a−c^Means of a row with no common superscript are significantly different (*P* < 0.05).

### 3.4 Antioxidant capacity

The antioxidant capacity results are shown in Table 5. For serum, dietary *B. laterosporus* decreased MDA concentrations, but linearly increased serum T-AOC activity (*P* < 0.01). Supplementation with MBL and HBL increased serum GSH-PX (*P* < 0.01). For liver, HBL increased hepatic T-AOC (*P* < 0.05). However, treatment did not affect hepatic GSH-PX, MDA, SOD, or CAT concentration. For breast muscle, SOD levels increased in the MBL group compared to CON group (*P* < 0.01). The HBL group had lower MDA concentrations than the CON group (*P* < 0.05).

**TABLE 5:** Effect of *B. laterosporus* supplementation on antioxidant indexes of broilers.

Items		Group				SEM	*P*-value
		CON	LBL	MBL	HBL		
Serum	T-AOC(U/mL)	1.35^Bc^	1.36^Ab^	1.37^Aab^	1.37^Aa^	0.001	0.0001
	SOD(pg/mL)	181.28	152.69	169.41	165.41	5.07	0.26
	GSH-PX(ng/mL)	13.14^Bb^	13.87^ABb^	14.76^Aa^	14.81^Aa^	0.19	0.0001
	CAT(pg/mL)	1457.78	1432.04	1454.80	1512.42	22.65	0.70
	MDA(mmol/L)	8.24^Aa^	6.94^Bb^	5.86^BCb^	6.47^Cc^	0.18	0.0001
Liver	T-AOC(U/mL)	2.04^b^	2.05^ab^	2.06^ab^	2.07^a^	0.001	0.03
	SOD(pg/mL)	1320.26^ab^	1241.71^b^	1434.83^a^	1205.46^b^	31.58	0.04
	GSH-PX(ng/mL)	9.62	10.69	11.42	7.85	0.70	0.30
	CAT(pg/mL)	1057.75	1069.68	1029.31	1029.54	20.13	0.87
	MDA(mmol/L)	5.33	5.24	5.00	4.94	0.13	0.68
Muscle	T-AOC(U/mL)	5.38	6.08	5.79	5.94	0.19	0.60
	SOD(pg/mL)	964.56^Bb^	1090.86^ABb^	1345.81^Aa^	1153.38^ABab^	40.25	0.0001
	GSH-Px(ng/mL)	10.12	10.52	10.23	9.58	0.19	0.36
	CAT(pg/mL)	1070.19	1031.14	974.93	999.77	20.09	0.38
	MDA(mmol/L)	5.56^ABa^	5.41^AB^	5.81^A^	4.91^Bb^	0.12	0.05

CAT, catalase; GSH-PX, glutathione peroxidase; MDA, malondialdehyde; SOD, superoxide dismutase; T-AOC, total antioxidative capacity. ^A−C^Means of a row with no common superscript are significantly different (*P* < 0.01). ^a−c^Means of a row with no common superscript are significantly different (*P* < 0.05).

### 3.5 Effects of *B. laterosporus* on microbial composition of the cecum

**FIGURE 1:**
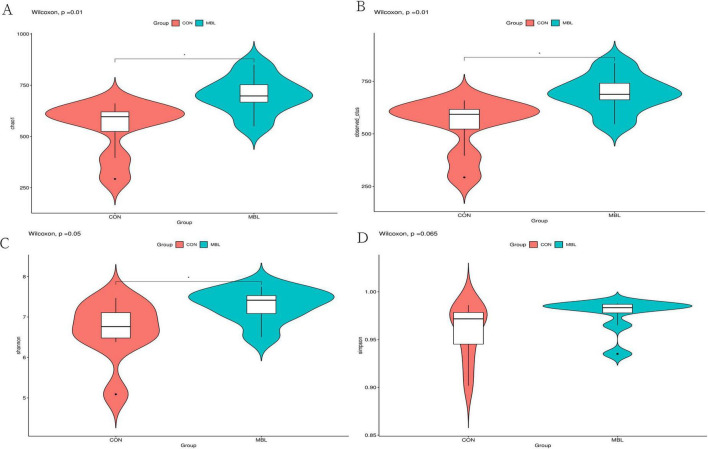
The diversity analysis of cecal microorganism. **(A–D)** The comparison of Chao 1, observed species, Shannon, and Simpson index between the CON and MBL groups.

According to the pre-production performance results, microbiological analysis was performed on cecum contents of the MBL and CON groups. Sequencing of 16S rDNA was performed using 16 samples to generate a total of 1,308,623 raw sequence reads. After the quality filter, there were 1,117,464 sequences (Supplementary Table S2). The rarefaction curves based on the Chao1 index approached an asymptote for each group, indicating that sufficient sequencing depth was achieved to adequately represent each microbiome community. This finding was further corroborated by complementary analyses using the Shannon index and observed OTUs (Supplementary Figure S1). Amplicon Sequence Variants (ASV) analysis showed that 2,882 ASVs were obtained in the MBL and CON groups. There were 1,087 similar ASVs between the two groups, 1,100 ASVs unique to MBL, and 685 ASVs unique to CON (Supplementary Figure S2). The cecum microbial composition analysis showed that compared with the CON group, the Shannon, Chao1, and observed species indexes in the MBL group were significantly higher (*P* < 0.05; Figures 1A–C). The Simpson index also tended to be higher in the MBL group than in the CON group (*P* = 0.065; Figure 1D).

**FIGURE 2:**
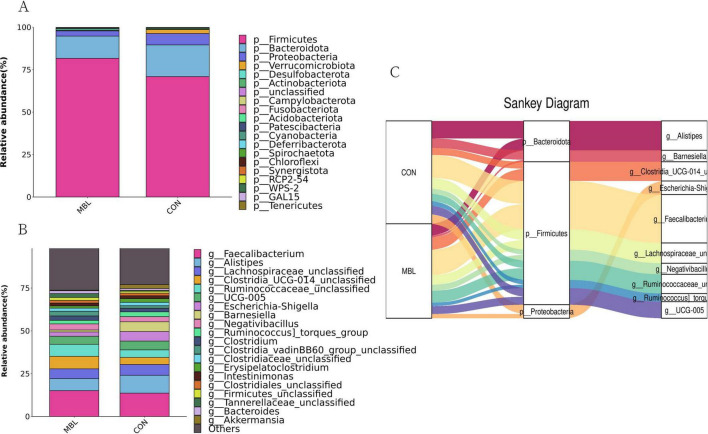
Relative abundance of the top phyla **(A)** and genus **(B)** in the samples. Sankey diagram of species composition at phylum and genus levels **(C)**.

**FIGURE 3:**
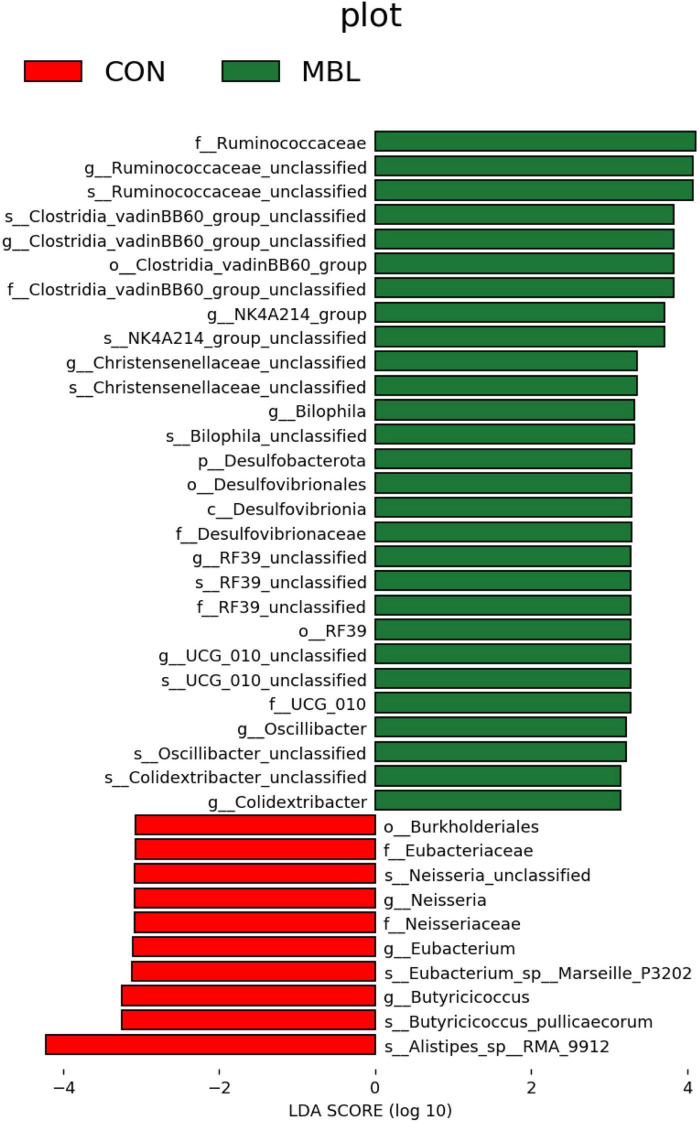
Histogram of the results of LEfSe among the MBL group and the CON group and their respective effect sizes.

Figure 2A shows that *Firmicutes*, *Bacteroidota*, and *Proteobacteria* were the most predominant phyla in each group, which together accounted for more than 90% of the total microbial community detected. At the genus level, *Faecalibacterium*, *Alistipes*, *Lachnospiraceae_unclassified*, *Clostridia_UCG-014_unclassified*, *Ruminococcaceae_unclassified*, *UCG-005, Escherichia-Shigella*, *Barnesiella*, *Negativibacillus*, *Ruminococcus_torques_group* were the most predominant in each group (Figure 2B). These species mainly belong to *Firmicutes*, *Bacteroidota*, and *Proteobacteria* (Figure 2C). LEfSe analysis was used to find biomarkers with statistical differences between the groups. Figure 3 shows the species with significant differences between CON and MBL groups with LDA > 3.0. *Ruminococcaceae_unclassified*, *Clostridia_vadinBB60_group_unclassified*, *NK4A214_group*, *Christensenellaceae_unclassified*, *Bilophila*, *RF39_unclassified*, *UCG-010_unclassified*, *Oscillibacter*, and *Colidextribacter* were enriched in the MBL group. *Eubacterium*, *Neisseria*, and *Butyriclcoccus* were more abundant in the CON group.

### 3.6 Effects of *B. laterosporus* on cecal metabolites

**FIGURE 4:**
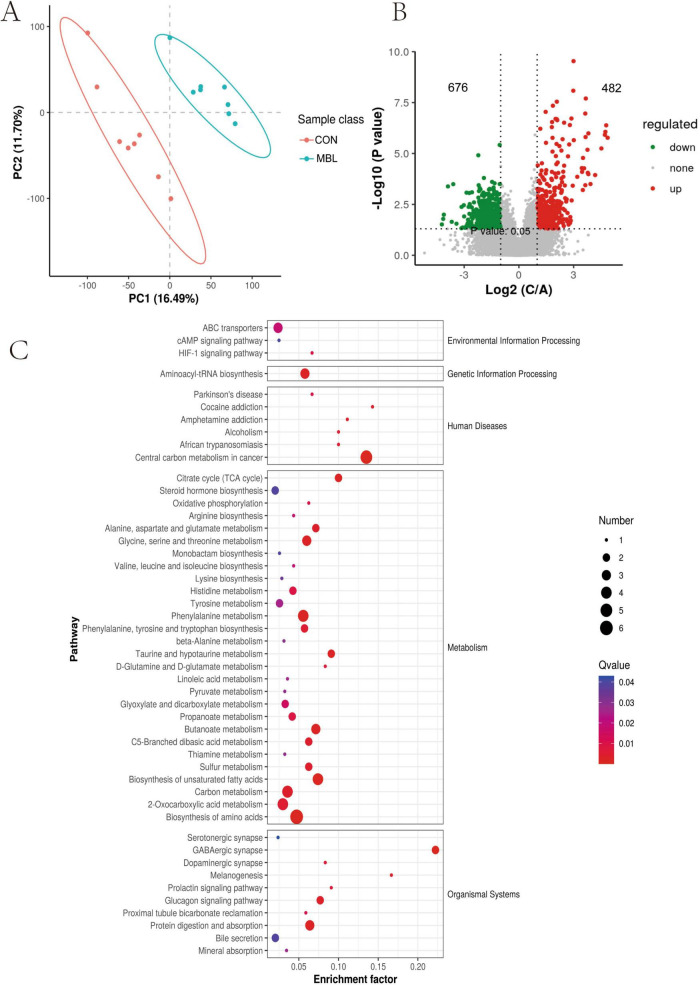
Identification of the metabolic signatures between the MBL group and the CON group. **(A)** Partial least squares discrimination analysis, **(B)** Volcano plot showed the DEMs; **(C)** KEGG analysis of the DEMs.

We identified a total of 23,717 peaks, including 12,357 primary metabolites and 1,906 secondary metabolites that were annotated. The PLS-DA model demonstrated a clear separation of metabolites among the different groups (Figure 4A). As illustrated in Figure 4B, 1,158 differentially expressed metabolites (DEMs) were identified between the MBL group and the CON group (Supplementary Table S3), only 103 had secondary metabolite annotation information. The DEMs in the cecum primarily included carboxylic acids and derivatives, fatty acyls, benzene and substituted derivatives, indoles and derivatives, organooxygen compounds, prenol lipids, and sterol lipids. The top 10 upregulated metabolites were aminocyclopyrachlor, nivalenol, 1,5-Isoquinolinediol, 3-Methoxy-4,5-methylenedioxybenzoic acid, 3-Oxopentanoic acid, O-Benzyl-L-serine, mono-(2-ethyl-5-hydroxyhexyl) phthalate, phosphohydroxypyruvic acid, citalopram, 2-Oxopentanedioic acid. Conversely, the top 10 downregulated metabolites were styrene, phenylethylamine, FAHFA 22:0; FAHFA (4:0/18:0), N-Acetyl-L-cysteine, taurine, purine, acylcarnitine 18:3, 7Z,10Z-Hexadecadienoic acid, N-Stearoyltaurine, hypoxanthine (Table 6). These DEMs were found to be mainly involved in metabolic pathways, including the biosynthesis of amino acids, 2-oxocarboxylic acid metabolism, carbon metabolism, biosynthesis of unsaturated fatty acids, phenylalanine metabolism, butanoate metabolism, protein digestion and absorption, and the glucagon signaling pathway (Figure 4C).

**TABLE 6:** Top 20 significantly different metabolites between the CON and MBL groups.

Metabolite	RT	Ratio	VIP	Class	*P* value	Regulated
Aminocyclopyrachlor	0.814	2.529	1.880	–	0.037	Up
Nivalenol	3.512	2.536	1.605	Prenol lipids	0.049	Up
1,5-Isoquinolinediol	3.940	2.597	1.808	Quinolines and derivatives	0.048	Up
3-Methoxy-4,5-methylenedioxybenzoic acid	0.813	2.709	2.097	Benzene and substituted derivatives	0.001	Up
3-Oxopentanoic acid	1.864	2.837	2.174	Keto acids and derivatives	0.001	Up
O-Benzyl-L-serine	0.814	3.492	2.275	–	0.001	Up
Mono-(2-ethyl-5-hydroxyhexyl) phthalate	4.486	4.099	2.061	Benzene and substituted derivatives	0.016	Up
Phosphohydroxypyruvic acid	0.742	4.114	2.753	Organooxygen compounds	0.0001	Up
Citalopram	1.413	4.364	2.854	Benzene and substituted derivatives	0.0001	Up
2-Oxopentanedioic acid	0.759	5.064	2.689	Keto acids and derivatives	0.001	Up
Styrene	3.486	0.056	2.395	Benzene and substituted derivatives	0.016	Down
Phenylethylamine	3.491	0.078	2.181	Benzene and substituted derivatives	0.023	Down
FAHFA 22:0; FAHFA (4:0/18:0)	8.993	0.167	2.301	Fatty Acyls	0.004	Down
N-Acetyl-L-cysteine	1.397	0.207	2.181	Carboxylic acids and derivatives	0.009	Down
Taurine	0.703	0.226	2.582	Organic sulfonic acids and derivatives	0.0001	Down
Purine	2.852	0.232	2.847	Imidazopyrimidines	0.001	Down
Acylcarnitine 18:3	6.499	0.247	2.690	Fatty Acyls	0.001	Down
7Z,10Z-Hexadecadienoic acid	8.027	0.256	2.179	Fatty Acyls	0.008	Down
N-Stearoyltaurine	7.075	0.260	2.521	–	0.045	Down
Hypoxanthine	1.117	0.264	2.485	Imidazopyrimidines	0.002	Down

RT, retention time; VIP, importance projection of OPLS-DA model; Class, Class classification of secondary metabolites, *P*, the *P*-value of the *t*-test.

### 3.7 Correlation between differential metabolites and cecal microbes

**FIGURE 5:**
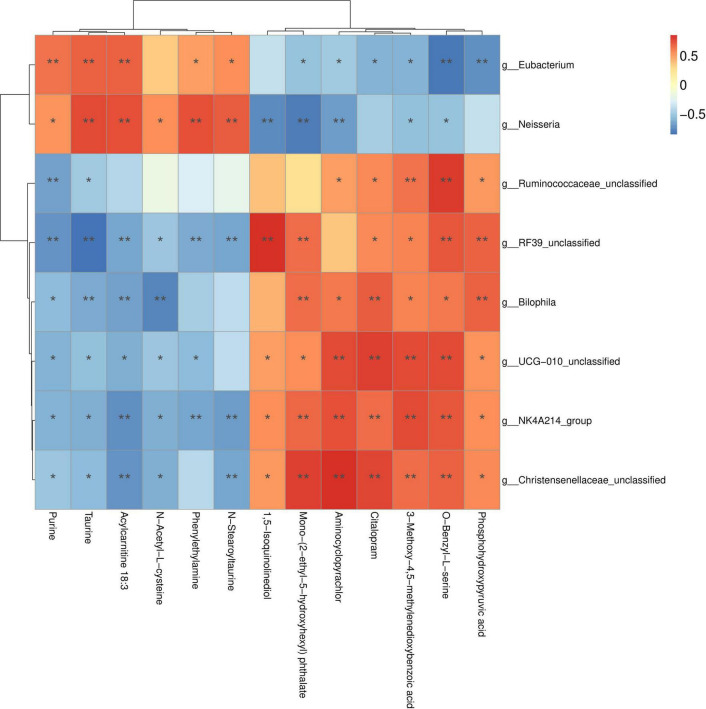
Correlations between differential metabolites and microbial genera.

Furthermore, Pearson’s correlation analysis revealed significant associations between the DEMs and eight distinct microbial genera (Figure 5), highlighting differences between the MBL and CON groups. Specifically, higher relative abundances of *Christensenellaceae_unclassified*, *NK4A214_group*, *Clostridia_UCG-010_unclassified*, *Bilophila, RF39_unclassified*, and *Ruminococcaceae_unclassified* were positively correlated with elevated concentrations of microbial metabolites in the MBL group, such as phosphohydroxypyruvic acid, O-benzyl-L-serine, 3-methoxy-4,5-methylenedioxybenzoic acid, citalopram, aminocyclopyrachlor, mono-(2-ethyl-5-hydroxyhexyl) phthalate, and 1,5-isoquinolinediol. In contrast, higher relative abundances of *Neisseria* and *Eubacterium* were positively associated with increased concentrations of stearoyltaurine, phenylethylamine, N-acetyl-L-cysteine, acylcarnitine 18:3, taurine, and purine in the CON group.

## 4 Discussion

We used YS909, a new hybrid of small broilers, to evaluate an antibiotic alternative. *B. laterosporus*, which has adequate thermal tolerance and acid and bile salt resistance (Liu Y. et al., 2024), represents a promising antibiotic alternative. Therefore, we investigated the effects of *B. laterosporus* on YS909 broilers. Growth performance and carcass trait characteristics are important factors when evaluating the economic benefits in broiler production. As a dietary additive, *B. laterosporus S62-9* can significantly promote the growth performance of broilers (Zhi et al., 2024). Consistent with previous studies, our results showed that LBL and MBL significantly decreased FCR. Liu et al. (2023) noted that the inclusion of *B. laterosporus S62-9* in feed resulted in increased breast muscle yield without significant changes in dressing, semi-eviscerated, eviscerated, or abdominal fat percentages. Conversely, our study demonstrated that dietary supplementation with *B. laterosporus* enhanced both semi-eviscerated and eviscerated percentages.

The evaluation of chicken meat quality in this study was based on pH, color, and shear force metrics. Our findings reveal that the inclusion of *B. laterosporus* in the diet did not markedly affect the shear force, pH levels, or coloration of the meat. These results align with studies on broilers fed *B. subtilis B2A*, which likewise reported no substantial effects on pH values or color attributes of meat (Park et al., 2020). However, Liu et al. (2023) reported a contrasting outcome, noting that the addition of *B. laterosporus S62-9* to the diet notably decreased shear force and enhanced the brightness (L*) and redness (a*) of the breast muscle. Therefore, the effect of probiotics on meat quality may be related to the types of probiotics.

Fatty acids, including UFA and SFA varieties, play a pivotal role in determining meat quality, nutritional value, and distinctive taste. Key fatty acids in chicken meat—namely, palmitic acid (C16:0), stearic acid (C18:0), oleic acid (C18:1n9c), and linoleic acid (C18:2n6c)—were consistent with our study’s observations (Liu X. et al., 2024). The introduction of *B. laterosporus* in our research significantly increased C18:3n3 levels while decreasing the proportions of C18:1n9t and C20:4n6. Studies on Chinese Jingxing Yellow chickens revealed a significant positive correlation between α-linolenic acid (C18:3n3) and total aldehydes and hexanal in chicken meat (Yuan et al., 2022). Aldehydes, the predominant volatile organic compounds (VOCs) in indigenous Chinese chicken breeds, contribute key aroma notes such as green grass, fatty, citrus, and bitter almond aromas to chicken meat, with hexanal being the most abundant (Jin et al., 2021). The trans configuration of C18:1n9t may contribute to a “hardened” texture or slight metallic off-flavors in meat. Additionally, elevated levels of this fatty acid can reduce juiciness and sweetness by altering lipid oxidation pathways (Wood et al., 2008). C20:4n6 (arachidonic acid) generates volatile aldehydes through lipid peroxidation, which contribute to “meaty” aroma and umami taste at low concentrations, but can lead to rancid off-flavors when present in excess (Zhang et al., 2022). Consequently, these results suggest *B. laterosporus* may alter meat flavor profiles through modifications of the muscular fatty acid profile.

Reactive oxygen species produced during normal cellular activities play important roles in various physiological processes; however, an imbalance in reactive oxygen species may damage DNA, proteins, and lipids, thereby negatively impacting growth performance and product quality (Estévez, 2015; Sies and Jones, 2020; Surai et al., 2019). Therefore, antioxidant balance is vital for normal physiological and metabolic functions in animals (Pang et al., 2021). Dietary probiotics reduce the damage caused by oxidative stress and promote antioxidant enzyme activity (Ji et al., 2022; Salem et al., 2022). In our study, *B. laterosporus* improved antioxidant capacity in YS909 broilers by increasing serum GSH-PX and T-AOC levels, and reducing MDA levels. Therefore, *B. laterosporus* has beneficial effects on the antioxidant capacity of YS909 broilers. In agreement with our findings, *Brevibacillus* improved antioxidant capacity in laying hens (Obianwuna et al., 2022). SOD, GST, GSH-PX, and CAT levels increased and MDA levels decreased. Previous study showed that antioxidant supplements protect against tissue damage by preventing the formation of radicals, by scavenging them, or by promoting their decomposition, resulting in better meat color (Falowo et al., 2014), however, our results showed that *B. laterosporus* supplementation only increased superoxide dismutase (SOD) activity in muscle, without significant effects on other antioxidant indices (e.g., GSH-Px, MDA). This targeted response implies that SOD induction alone may not sufficiently modulate the oxidative cascade to improve meat color stability.

Moreover, the microflora in the cecum is crucial for the health and growth performance of chickens, influencing the transformation of food, resistance to diseases, and the ability to ward off pathogenic colonization (Rychlik, 2020). To investigate the effect of *B. laterosporus* on cecal microorganisms, we sequenced 16S rDNA from the cecal content. Our results showed that the α-diversity indexes such as Shannon, Chao1, and observed species significantly increased after *B. laterosporus* supplementation. An increase in α-diversity promotes the productivity and barrier integrity of the intestinal epithelium (Cao et al., 2018). α-Diversity is related to F/G of animals, and the higher the α-diversity, the lower the F/G (Aliakbari et al., 2021). A previous study reported that supplementation with *Clostridium butyricum* enhanced the α-diversity of the intestinal microbiota and growth performance in Pekin ducks (Liu et al., 2021). In our study, *B. laterosporus* supplementation decreased the F/G and increased the semi-eviscerated and eviscerated percentages.

We examined the shifts in the cecal microbial community at both phylum and genus levels based on species annotation. Predominantly, the microbial populations in each group were composed of *Firmicutes*, *Bacteroidota*, and *Proteobacteria* at the phylum level, collectively representing over 90% of the detected total microbial community. This observation aligns with prior research identifying these three as key microbial communities in chicken guts at the phylum level (Zhang et al., 2021). Notably, the MBL group exhibited an increased relative abundance of *Firmicutes* in comparison to the CON group, whereas the proportions of *Bacteroidetes* and *Proteobacteria* were lower. *Firmicutes* play a crucial role in the metabolism and energy extraction processes of animals. Their heightened presence could potentially improve the breakdown and absorption of carbohydrates and fats, leading to enhanced energy assimilation (Hu et al., 2018). Animal growth performance has been observed to correlate positively with the presence of *Firmicutes*, particularly in terms of the ratio of *Firmicutes* to *Bacteroidetes* in the gut (Crisol-Martínez et al., 2017; Han et al., 2017). The elevated *Firmicutes*-to-*Bacteroidetes* ratio observed in the MBL group suggests an increased *Firmicutes* count and a decreased *Bacteroidetes* count, offering a plausible explanation for the observed improvements in semi-eviscerated and eviscerated percentages in broilers within the MBL group. At the genus level, the dominant bacteria species of the cecum were *Faecalibacterium*, *Alistipes*, *Lachnospiraceae_unclassified*, *Clostridia_UCG-014_unclassified*, and *Ruminococcaceae_unclassified*. These findings are in agreement with a previous study, where the main bacterial genera identified in the chicken cecum were *Clostridium*, *Ruminococcus*, *Lactobacillus*, *Bacteroides*, *Alistipes*, and *Faecalibacterium* (Alfaia et al., 2021). LEfSe analysis identified more representative species as biomarkers to distinguish the microbiota of the MBL and CON groups. *Ruminococcaceae_unclassified*, *Clostridia_vadinBB60_group_unclassified*, *NK4A214_group*, *Christensenellaceae_unclassified*, *Bilophila*, *RF39_unclassified*, *UCG-010_unclassified*, *Oscillibacter*, and *Colidextribacter* were enriched in the MBL group, while *Eubacterium*, *Neisseria*, and *Butyriclcoccus* were more abundant in the CON group. *Rumenococcus* and *Clostridia* are common bacteria in the cecum and belong to *Firmicutes* and mostly butyrate producers (Parada Venegas et al., 2019; Yang et al., 2017). The addition of butyrate to the animal diet is beneficial in improving growth performance (Venardou et al., 2021). A reduction in *Christensenellaceae* in the gut is associated with gut inflammation (Kennedy et al., 2018). *Neisseria* is a symbiotic bacterium of human and animal mucous membranes, which can cause a variety of diseases in humans and animals (Li et al., 2022; York, 2021). The increased abundance in *Ruminococcaceae_unclassified*, *Clostridia_vadinBB60_group_unclassified*, and *Christensenellaceae_unclassified* and a reduction in the abundance of *Neisseria* in cecum may contribute to the improvement of performance and health of broilers supplemented with *B. laterosporus*.

We also investigated the effects of *B. laterosporus* on cecal metabolites in broilers. A total of 1,158 DEMs were identified between the MBL group and the CON group. These DEMs were primarily involved in amino acid and lipid metabolism. Consistent with our findings, Cao et al. (2018) reported that supplementing chickens’ diets with *B. amyloliquefaciens* altered the levels of gut metabolites associated with amino acid and glyceride metabolism. In the present study, metabolites such as 2-oxopentanedioic acid, citalopram, N-ethylglycine, phosphohydroxypyruvic acid, and mono-(2-ethyl-5-hydroxyhexyl) phthalate were significantly upregulated in the MBL group. 2-Oxopentanedioic acid (also known as alpha-ketoglutaric acid) plays a critical role in cellular energy metabolism as an intermediate in the tricarboxylic acid (TCA) cycle, which is essential for the oxidation of fatty acids, amino acids, and glucose. Recent studies have also highlighted its additional functions, including modulation of the immune response and maintenance of intestinal homeostasis (Harrison and Pierzynowski, 2008). N-ethylglycine, another key metabolite, serves as an important intermediate in energy metabolism and amino acid biosynthesis. Previous studies have shown that dietary supplementation with *L. paracasei ZFM54* and *B. subtilis* increased the levels of N-ethylglycine in the cecum (Chen et al., 2024; Park et al., 2020). Phosphohydroxypyruvic acid, an intermediate in glycolysis and amino acid synthesis, also plays a regulatory role in enzyme activity within metabolic pathways (Borisenko et al., 2022). The observed changes in cecal metabolites may be attributed to alterations in the gut microbiota. To explore this, we analyzed the correlations between metabolites and microbial genera. The results revealed that phosphohydroxypyruvic acid and O-benzyl-L-serine were positively correlated with *Christensenellaceae_unclassified*, *NK4A214_group*, *Clostridia_UCG-010_unclassified*, *Bilophila*, *RF39_unclassified*, and *Ruminococcaceae_unclassified*. In contrast, acylcarnitine 18:3, taurine, and purine were positively correlated with *Neisseria* and *Eubacterium*.

## 5 Conclusion

Dietary supplementation with medium-dose *B. laterosporus* modulated the cecal microbiota of broilers by significantly increasing the relative abundance of *Christensenellaceae_ unclassified*, *NK4A214_group*, *Clostridia_UCG-010_unclassified*, *Bilophila, RF39_unclassified*, and *Ruminococcaceae_unclassified* This microbial shift consequently elevated the concentrations of key metabolites involved in the TCA cycle and amino acid metabolism, including phosphohydroxypyruvic acid, O-benzyl-L-serine, 3-methoxy-4,5-methylenedioxybenzoic acid, aminocyclopyrachlor, and mono-(2-ethyl-5-hydroxyhexyl) phthalate. These metabolic alterations ultimately led to changes in nutrient absorption profile, thereby improving production performance in broilers.

## Data Availability

The Genome Sequence Archive in the National Genomics Data Center, a part of the China National Center for Bioinformation/Beijing Institute of Genomics, Chinese Academy of Sciences (GSA: CRA013160), archived the raw sequence data, now accessible at https://ngdc.cncb.ac.cn/gsa.

## Supplementary material

Supplementary Figure S1 Rarefaction curve for CON and MBL groups. **(A)** Chao 1, **(B)** Shannon index, **(C)** observed OTUs.

Supplementary Figure S2 Venn diagram of amplicon sequence variants (ASV) between the CON and MBL groups.

## References

[B1] Alfaia C. Pestana J. Rodrigues M. Coelho D. Aires M. Ribeiro D. (2021). Influence of dietary *Chlorella vulgaris* and carbohydrate-active enzymes on growth performance, meat quality and lipid composition of broiler chickens. *Poult. Sci.* 100 926–937. 10.1016/j.psj.2020.11.034 33518146 PMC7858185

[B2] Aliakbari A. Zemb O. Billon Y. Barilly C. Ahn I. Riquet J. (2021). Genetic relationships between feed efficiency and gut microbiome in pig lines selected for residual feed intake. *J. Anim. Breed. Genet.* 138 491–507. 10.1111/jbg.12539 33634901 PMC8248129

[B3] Andreou L. (2013). Preparation of genomic DNA from bacteria. *Methods Enzymol.* 529 143–151. 10.1016/B978-0-12-418687-3.00011-2 24011042

[B4] Bolyen E. Rideout J. Dillon M. Bokulich N. Abnet C. Al-Ghalith G. (2019). Reproducible, interactive, scalable and extensible microbiome data science using QIIME 2. *Nat. Biotechnol.* 37 852–857. 10.1038/s41587-019-0209-9 31341288 PMC7015180

[B5] Borisenko S. Fedorov A. Kuibarov A. Bianchi M. Bezguba V. Majchrzak P. (2022). Fermi surface tomography. *Nat. Commun.* 13:4132. 10.1038/s41467-022-31841-z 35840603 PMC9287296

[B6] Callahan B. McMurdie P. Rosen M. Han A. Johnson A. Holmes S. (2016). DADA2: High-resolution sample inference from Illumina amplicon data. *Nat. Methods* 13 581–583. 10.1038/nmeth.3869 27214047 PMC4927377

[B7] Cao G. Zhan X. Zhang L. Zeng X. Chen A. Yang C. (2018). Modulation of broilers’ caecal microflora and metabolites in response to a potential probiotic *Bacillus amyloliquefaciens*. *J. Anim. Physiol. Anim. Nutr.* 102 e909–e917. 10.1111/jpn.12856 29314285

[B8] Che J. Ye S. Liu B. Deng Y. Chen Q. Ge C. (2016). Effects of *Brevibacillus brevis* FJAT-1501-BPA on growth performance, faecal microflora, faecal enzyme activities and blood parameters of weaned piglets. *Antonie Van Leeuwenhoek* 109 1545–1553. 10.1007/s10482-016-0756-8 27558133

[B9] Chen X. Zhu Z. Zhang X. Chen L. Gu Q. Li P. (2024). *Lactobacillus paracasei* ZFM54 alters the metabolomic profiles of yogurt and the co-fermented yogurt improves the gut microecology of human adults. *J. Dairy Sci.* 107 5280–5300. 10.3168/jds.2023-24332 38460876

[B10] Crisol-Martínez E. Stanley D. Geier M. Hughes R. Moore R. (2017). Understanding the mechanisms of zinc bacitracin and avilamycin on animal production: Linking gut microbiota and growth performance in chickens. *Appl. Microbiol. Biotechnol.* 101 4547–4559. 10.1007/s00253-017-8193-9 28243710

[B11] Edwards R. (2011). Quality control and preprocessing of metagenomic datasets. *Bioinformatics* 27 863–864. 10.1093/bioinformatics/btr026 21278185 PMC3051327

[B12] Estévez M. (2015). Oxidative damage to poultry: From farm to fork. *Poult. Sci.* 94 1368–1378. 10.3382/ps/pev094 25825786

[B13] Falowo A. Fayemi P. Muchenje V. (2014). Natural antioxidants against lipid–protein oxidative deterioration in meat and meat products: A review. *Food Res. Int.* 64 171–181. 10.1016/j.foodres.2014.06.022 30011637

[B14] Halder N. Sunder J. De Ak, Bhattacharya D. Joardar S. (2024). Probiotics in poultry: A comprehensive review. *J. Basic Appl. Zool.* 85:23. 10.1186/s41936-024-00379-5

[B15] Han G. Lee J. Jin G. Park J. Choi Y. Chae B. (2017). Evaluating the association between body weight and the intestinal microbiota of weaned piglets via 16S rRNA sequencing. *Appl. Microbiol. Biotechnol.* 101 5903–5911. 10.1007/s00253-017-8304-7 28523395

[B16] Harrison A. Pierzynowski S. (2008). Biological effects of 2-oxoglutarate with particular emphasis on the regulation of protein, mineral and lipid absorption/metabolism, muscle performance, kidney function, bone formation and cancerogenesis, all viewed from a healthy ageing perspective state of the art-review article. *J. Physiol. Pharmacol.* 59 91–106. 18802218

[B17] Hu L. Geng S. Li Y. Cheng S. Fu X. Yue X. (2018). Exogenous fecal microbiota transplantation from local adult pigs to crossbred newborn piglets. *Front. Microbiol.* 8:2663. 10.3389/fmicb.2017.02663 29375527 PMC5767267

[B18] Jamil M. Khatoon A. Saleemi M. Abbas R. (2025). *Bacillus licheniformis* as a protective agent in broiler chicken concurrently exposed to mycotoxins and necrotic enteritis: Toxicopathological and hematobiochemical perspectives. *Microb. Pathog.* 198:107108. 10.1016/j.micpath.2024.107108 39510360

[B19] Ji L. Zhang L. Liu H. Shen J. Zhang Y. Lu L. (2022). *Bacillus subtilis* M6 improves intestinal barrier, antioxidant capacity and gut microbial composition in AA broiler. *Front. Nutr.* 9:965310. 10.3389/fnut.2022.965310 36061900 PMC9428444

[B20] Jin Y. Cui H. Yuan X. Liu L. Liu X. Wang Y. (2021). Identification of the main aroma compounds in Chinese local chicken high-quality meat. *Food Chem.* 359:129930. 10.1016/j.foodchem.2021.129930 33951611

[B21] Kennedy N. Lamb C. Berry S. Walker A. Mansfield J. Parkes M. (2018). The impact of NOD2 variants on fecal microbiota in Crohn’s disease and controls without gastrointestinal disease. *Inflamm. Bowel Dis.* 24 583–592. 10.1093/ibd/izx061 29462388 PMC6176884

[B22] Li Y. A. Sun Y. Fu Y. Zhang Y. Li Q. Wang S. (2022). *Salmonella enterica* serovar Choleraesuis vector delivering a dual-antigen expression cassette provides mouse cross-protection against *Streptococcus suis* serotypes 2, 7, 9, and 1/2. *Vet. Res.* 53:46. 10.1186/s13567-022-01062-9 35733156 PMC9215036

[B23] Liu J. Gu H. Jia R. Li S. Chen Z. Zheng A. (2025). Effects of Lactobacillus acidophilus on production performance and immunity of broiler chickens and their mechanism. *Front. Vet. Sci.* 12:1554502. 10.3389/fvets.2025.1554502 40196813 PMC11974341

[B24] Liu X. Ma A. Zhi T. Hong D. Chen Z. Li S. (2023). Dietary effect of *Brevibacillus laterosporus* S62-9 on chicken meat quality, amino acid profile, and volatile compounds. *Foods* 12:288. 10.3390/foods12020288 36673380 PMC9858446

[B25] Liu X. Wang Y. Wang Y. Cui H. Zhao G. Guo Y. (2024). Effect of myristic acid supplementation on triglyceride synthesis and related genes in the pectoral muscles of broiler chickens. *Poult. Sci.* 103:104038. 10.1016/j.psj.2024.104038 39079330 PMC11340564

[B26] Liu Y. Liu C. An K. Gong X. Xia Z. (2021). Effect of dietary *Clostridium butyricum* supplementation on growth performance, intestinal barrier function, immune function, and microbiota diversity of Pekin ducks. *Animals* 11:2514. 10.3390/ani11092514 34573480 PMC8471152

[B27] Liu Y. Zai X. Weng G. Ma X. Deng D. (2024). *Brevibacillus laterosporus*: A probiotic with important applications in crop and animal production. *Microorganisms* 12:564. 10.3390/microorganisms12030564 38543615 PMC10975594

[B28] Logue J. Stedmon C. Kellerman A. Nielsen N. Andersson A. Laudon H. (2016). Experimental insights into the importance of aquatic bacterial community composition to the degradation of dissolved organic matter. *ISME J.* 10 533–545. 10.1038/ismej.2015.131 26296065 PMC4817675

[B29] Ma F. Xu S. Tang Z. Li Z. Zhang L. (2021). Use of antimicrobials in food animals and impact of transmission of antimicrobial resistance on humans. *Biosaf. Health* 3, 32–38. 10.1016/j.bsheal.2020.09.004

[B30] Magoč T. Salzberg S. (2011). FLASH: Fast length adjustment of short reads to improve genome assemblies. *Bioinformatics* 27 2957–2963. 10.1093/bioinformatics/btr507 21903629 PMC3198573

[B31] Muhammad J. Khan S. Su J. Q. Hesham A. E.-L. Ditta A. Nawab J. (2020). Antibiotics in poultry manure and their associated health issues: A systematic review. *J. Soils Sediments* 20, 486–497. 10.1007/s11368-019-02360-0

[B32] Obianwuna U. Qiu K. Chang X.-Y. Zhang H.-J. Wang J. Qi G.-H. (2022). Enhancing egg production and quality by the supplementation of probiotic strains (*Clostridium* and *Brevibacillus*) via improved amino acid digestibility, intestinal health, immune response, and antioxidant activity. *Front. Microbiol.* 13:987241. 10.3389/fmicb.2022.987241 36177461 PMC9512665

[B33] Pang B. Chan W. Chan C. (2021). Mitochondria homeostasis and oxidant/antioxidant balance in skeletal muscle—do myokines play a role? *Antioxidants* 10:179. 10.3390/antiox10020179 33513795 PMC7911667

[B34] Parada Venegas D. De la Fuente M. K. Landskron G. González M. J. Quera R. Dijkstra G. (2019). Short chain fatty acids (SCFAs)-mediated gut epithelial and immune regulation and its relevance for inflammatory bowel diseases. *Front. Immunol.* 10:277. 10.3389/fimmu.2019.00277 30915065 PMC6421268

[B35] Park I. Zimmerman N. Smith A. Rehberger T. Lillehoj E. Lillehoj H. (2020). Dietary supplementation with *Bacillus subtilis* direct-fed microbials alters chicken intestinal metabolite levels. *Front. Vet. Sci.* 7:123. 10.3389/fvets.2020.00123 32195276 PMC7064633

[B36] Purba M. Sepriadi S. Trisna A. Desnamrina K. Hua L. (2022). “The effect of *Brevibacillus laterosporus* texasporus culture on percentage of carcass broilers chickens infected with *Salmonella pullorum*,” in *Proceedings of the IOP conference series: Earth and environmental science*, (Bristol: IOP Publishing), 10.1088/1755-1315/977/1/012133

[B37] Quast C. Pruesse E. Yilmaz P. Gerken J. Schweer T. Yarza P. (2013). The SILVA ribosomal RNA gene database project: Improved data processing and web-based tools. *Nucleic Acids Res.* 41 D590–D596. 10.1093/nar/gks1219 23193283 PMC3531112

[B38] Rognes T. Flouri T. Nichols B. Quince C. Mahé F. (2016). VSEARCH: A versatile open source tool for metagenomics. *PeerJ.* 4:e2584. 10.7717/peerj.2584 27781170 PMC5075697

[B39] Rychlik I. (2020). Composition and function of chicken gut microbiota. *Animals* 10:103. 10.3390/ani10010103 31936291 PMC7022619

[B40] Salem H. Alqhtani A. Swelum A. Babalghith A. Melebary S. Soliman S. (2022). Heat stress in poultry with particular reference to the role of probiotics in its amelioration: An updated review. *J. Therm. Biol.* 108:103302. 10.1016/j.jtherbio.2022.103302 36031223

[B41] Sies H. Jones D. (2020). Reactive oxygen species (ROS) as pleiotropic physiological signalling agents. *Nat. Rev. Mol. Cell Biol.* 21 363–383. 10.1038/s41580-020-0230-3 32231263

[B42] Smith C. Want E. O’Maille G. Abagyan R. Siuzdak G. (2006). XCMS: Processing mass spectrometry data for metabolite profiling using nonlinear peak alignment, matching, and identification. *Anal. Chem.* 78 779–787. 10.1021/ac051437y 16448051

[B43] Surai P. Kochish I. Fisinin V. Kidd M. (2019). Antioxidant defence systems and oxidative stress in poultry biology: An update. *Antioxidants* 8:235. 10.3390/antiox8070235 31336672 PMC6680731

[B44] Tian M. He X. Feng Y. Wang W. Chen H. Gong M. (2021). Pollution by antibiotics and antimicrobial resistance in livestock and poultry manure in China, and countermeasures. *Antibiotics* 10:539. 10.3390/antibiotics10050539 34066587 PMC8148549

[B45] Venardou B. O’Doherty J. Vigors S. O’Shea C. Burton E. Ryan M. (2021). Effects of dietary supplementation with a laminarin-rich extract on the growth performance and gastrointestinal health in broilers. *Poult. Sci.* 100:101179. 10.1016/j.psj.2021.101179 34098504 PMC8187820

[B46] Wang J. Yao L. Su J. Fan R. Zheng J. Han Y. (2023). Effects of *Lactobacillus plantarum* and its fermentation products on growth performance, immune function, intestinal pH, and cecal microorganisms of Lingnan yellow chicken. *Poult. Sci.* 102:102610. 10.1016/j.psj.2023.102610 37019072 PMC10106959

[B47] Wen B. Mei Z. Zeng C. Liu S. (2017). metaX: A flexible and comprehensive software for processing metabolomics data. *BMC Bioinform.* 18:183. 10.1186/s12859-017-1579-y 28327092 PMC5361702

[B48] Wolfenden R. Pumford N. Morgan M. Shivaramaiah S. Wolfenden A. Pixley C. (2011). Evaluation of selected direct-fed microbial candidates on live performance and *Salmonella* reduction in commercial turkey brooding houses. *Poult. Sci.* 90 2627–2631. 10.3382/ps.2011-01360 22010250

[B49] Wood J. Enser M. Fisher A. Nute G. Sheard P. Richardson R. (2008). Fat deposition, fatty acid composition and meat quality: A review. *Meat Sci.* 78 343–358. 10.1016/j.meatsci.2007.07.019 22062452

[B50] Yang L. Liu S. Ding J. Dai R. He C. Xu K. (2017). Gut microbiota co-microevolution with selection for host humoral immunity. *Front. Microbiol.* 8:1243. 10.3389/fmicb.2017.01243 28725219 PMC5495859

[B51] York A. (2021). A new general mechanism of AMR. *Nat. Rev. Microbiol.* 19 283–283. 10.1038/s41579-021-00539-2 33658650

[B52] Yuan X. Cui H. Jin Y. Zhao W. Liu X. Wang Y. (2022). Fatty acid metabolism-related genes are associated with flavor-presenting aldehydes in Chinese local chicken. *Front. Genet.* 13:902180. 10.3389/fgene.2022.902180 36035160 PMC9412053

[B53] Zhang D. Ivane N. Haruna S. Zekrumah M. Elyse F. Tahir H. (2022). Recent trends in the micro-encapsulation of plant-derived compounds and their specific application in meat as antioxidants and antimicrobials. *Meat Sci.* 191:108842. 10.1016/j.meatsci.2022.108842 35660290

[B54] Zhang S. Zhong G. Shao D. Wang Q. Hu Y. Wu T. (2021). Dietary supplementation with *Bacillus subtilis* promotes growth performance of broilers by altering the dominant microbial community. *Poult. Sci.* 100:100935. 10.1016/j.psj.2020.12.032 33652528 PMC7936199

[B55] Zhi T. Ma A. Liu X. Chen Z. Li S. Jia Y. (2024). Dietary supplementation of *Brevibacillus laterosporus* S62-9 improves broiler growth and immunity by regulating cecal microbiota and metabolites. *Probiotics Antimicrob. Proteins* 16 949–963. 10.1007/s12602-023-10088-0 37211578

[B56] Zou X. Zhang M. Tu W. Zhang Q. Jin M. Fang R. (2022). *Bacillus subtilis* inhibits intestinal inflammation and oxidative stress by regulating gut flora and related metabolites in laying hens. *Animal* 16:100474. 10.1016/j.animal.2022.100474 35220172

