# Supplementary material for: Effect of dietary supplementation with Brevibacillus laterosporus on broiler growth performance, meat quality and gut microbiome
Source: Front Microbiol. 2025 Jun 18;16:1608076. doi: 10.3389/fmicb.2025.1608076 (PMC12213765; doi:10.3389/fmicb.2025.1608076)
Supplement: Supplementary file 1 [file Table_1.docx]

**Table S1 Basic diet and nutrient composition of experimental broilers.**

| Item | Contents | | |
| --- | --- | --- | --- |
|  | 1 to 21days old | | 22-42 days old |
| Ingredients（%） |  |  | |
| Corn | 63.77 | 67.90 | |
| Soybean meal | 27.85 | 21.37 | |
| Wheat bran | 0.50 | 1.50 | |
| Corn gluten meal | 4.30 | 5.00 | |
| Limestone | 0.96 | 0.76 | |
| CaHPO_4_ | 1.80 | 1.75 | |
| Soybean oil | 0.00 | 0.90 | |
| Salt | 0.32 | 0.32 | |
| Premix^1^ | 0.50 | 0.50 | |
| Nutrient levels^2^ | | | |
| ME（MJ/kg） | 2913.13 | 3011.27 | |
| CP（%） | 21.02 | 19.02 | |
| Met+Cys（%） | 0.85 | 0.72 | |
| Thr（%） | 0.76 | 0.74 | |
| Lys（%） | 1.05 | 0.98 | |
| Ca（%） | 1.00 | 0.90 | |
| P（%） | 0.68 | 0.65 | |

^1^The premix provided the following per kg of diets: VA 6,000 IU, VD3 1,000 IU, VE 15 IU, VK3 0.5 mg, VB1 2 mg, VB2 4 mg, D-pantothenic acid 10 mg, nicotinic acid 35 mg, VB6 3.5 mg, VB12 0.01mg, biotin 0.18 mg, folic acid 0.55mg, Cu (as copper sulfate) 8 mg, Fe (as ferrous sulfate) 90 mg, Mn (as manganese sulfate) 90 mg, Zn (as zinc sulfate) 650 mg, Se (as sodium selenite) 0.20 mg.

^2^The nutrient levels were measured values.
