# Supplementary figures and images for: Effect of dietary supplementation with Brevibacillus laterosporus on broiler growth performance, meat quality and gut microbiome
Source: Front Microbiol. 2025 Jun 18;16:1608076. doi: 10.3389/fmicb.2025.1608076 (PMC12213765; doi:10.3389/fmicb.2025.1608076)

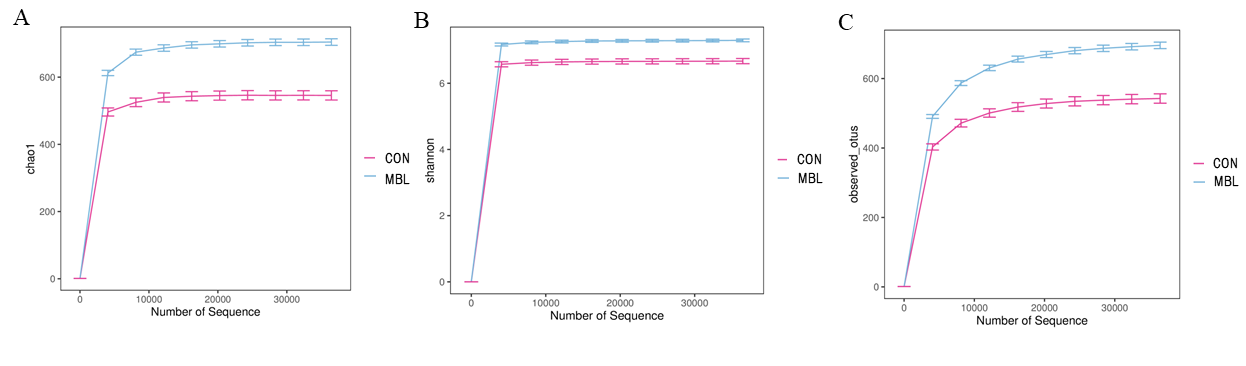

Supplement: Supplementary Figure S1 — Rarefaction curve for CON and MBL groups. (A) Chao 1, (B) Shannon index, (C) observed OTUs. [file Image_1.tif]

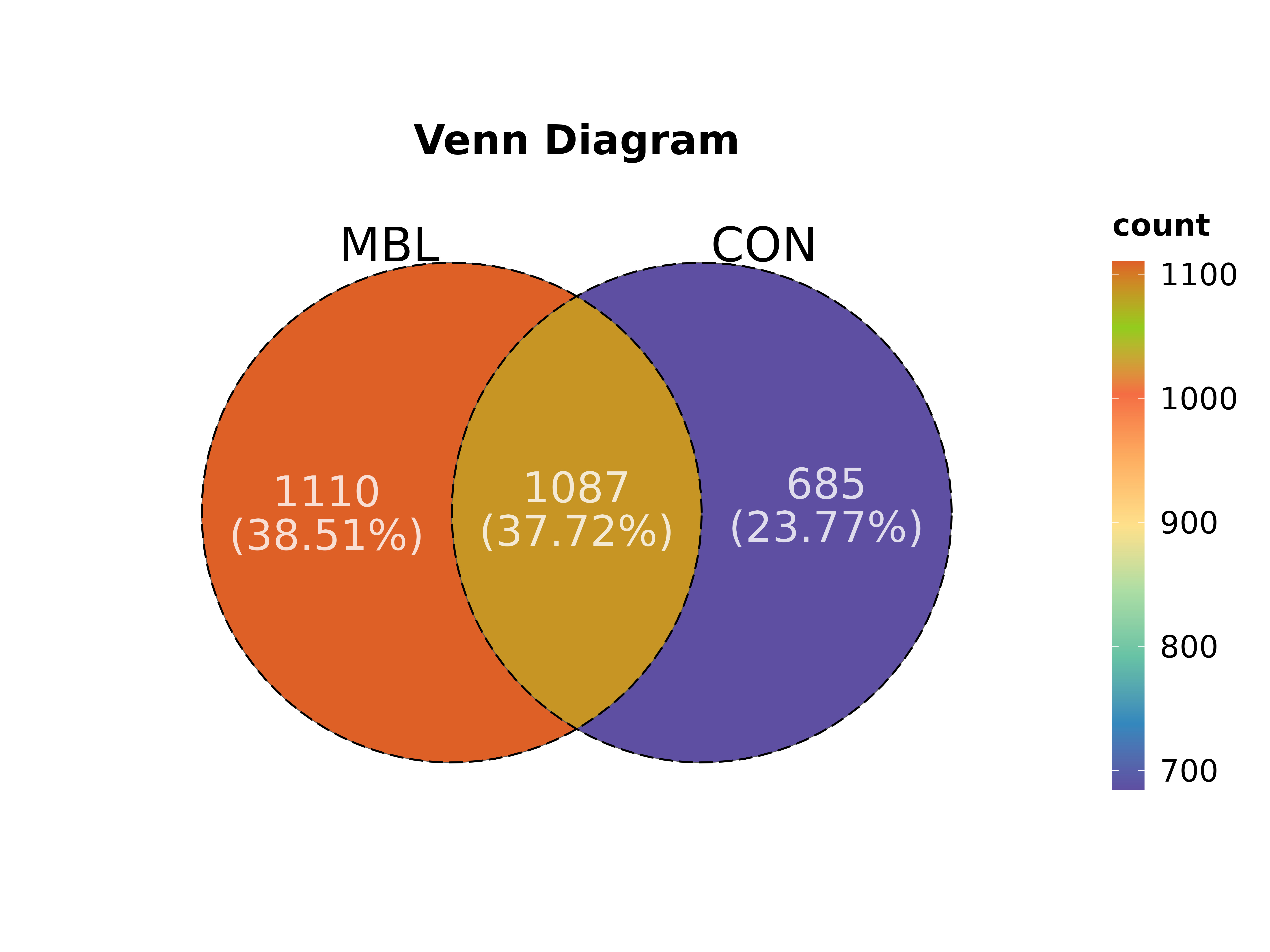

Supplement: Supplementary Figure S2 — Venn diagram of amplicon sequence variants (ASV) between the CON and MBL groups. [file Image_2.jpeg]
